# Bilateral pelvic kidneys with upper pole fusion and malrotation: a  case report and  review of the literature

**DOI:** 10.1186/s13256-021-02761-1

**Published:** 2021-04-05

**Authors:** Hussam S. Khougali, Omer Ali Mohamed Ahmed Alawad, Nicholas Farkas, Mohammed Mahgoub Mirghani Ahmed, Alnasri Mohammed Abuagla

**Affiliations:** 1 Western Sussex Hospitals NHS Trust, Chichester, UK; 2 Wad-Medani Teaching Hospital, Wad Madani, Sudan; 3 Department of General surgery, Western Sussex Hospitals, Chichester, UK; 4 Department of Radiology, Wad-Medani Teaching Hospital, Wad Madani, Sudan

**Keywords:** Bilateral pelvic kidneys, Renal ectopia, Renal reversed rotation

## Abstract

**Background:**

The incidence of ectopic kidneys is 1:12,000 clinically and 1:900 postmortem. Patients with pelvic mal-rotated kidneys are more susceptible to recurrent urinary tract infections, recurrent renal stones, and renal injury. Fusion of the kidney lower poles is relatively common compared to other types of renal anomalies.

**Case presentation:**

We present the case of a 36-year-old Sudanese female patient who presented with a long history of recurrent urinary tract infections unresponsive to antibiotics. Ultrasound scan revealed bilateral pelvic kidneys. Computed tomography (CT) urography confirmed bilateral ectopic fused kidneys, with the left kidney mal-rotated (renal pelvis facing upwards and laterally). Kidney infection secondary to vesicoureteral reflux was diagnosed. Antibiotics were prescribed according to culture and sensitivity. The patient responded well to ciprofloxacin.

**Conclusion:**

A history of recurrent urinary tract infections without an apparent cause is highly suggestive of renal anomaly and should be investigated expediently. Ultrasonography or CT imaging may be utilized to aid in diagnosis. Early recognition may help prevent the high risk of end-stage renal failure associated with anomalies.

## Background

The incidence of ectopic kidneys is 1:12,000 clinically and 1:900 postmortem [1, 2]. Patients with pelvic mal-rotated kidneys are more susceptible to recurrent unitary tract infections, recurrent renal stones, and renal injury; therefore, meticulous preventive measures should be observed [3]. Renal fusion anomaly is an abnormality of position, rotation, and migration. It is more frequent in males [4]. Renal fusion anomalies may result in significant difficulties during retroperitoneal interventions such as abdominal aortic aneurysm surgery, pelvic surgeries, and percutaneous nephrostomy or renal transplantation [4, 5].

The normal location of the kidneys is retroperitoneal on either side of the vertebral column at the level of the L2 vertebra. When the kidneys are not located in such positions, they are called ectopic kidneys [6]. The development of the urogenital system in humans is a complex process that starts in the pelvis, and by the ninth gestational week the kidneys reach their retroperitoneal position [7]. Pelvic kidneys can be unilateral or bilateral and fused or non-fused, and can also be found anywhere in the abdomen along their pathway of ascent [8]. In over 90% of cases, fusion between the kidneys occurs at the lower pole. However, it is not clear whether abnormal position or fusion results from anomalous renal blood supply. Upper pole fusion remains very rare [8]. We reviewed the literature by searching the PubMed database and Google Scholar using “pelvic kidneys AND upper pole fusion” and “horseshoe kidney AND upper pole fusion” as key words and found no such case reported. This kind of renal anomaly can be easy missed for long periods if there is no proper assessment. We present this rare case in order to raise awareness among clinicians regarding the importance of proper clinical assessment, investigation, and treatment of such abnormality to prevent complications that increase the risk of renal failure.

## Case presentation

A 36-year-old Sudanese female patient presented to Gezira Center of Nephrology and Urology, Sudan, complaining of burning micturition and back pain. A pregnancy test at the time of presentation was negative. A history of similar symptoms with increasing frequency over the preceding 2 years was reported.

The patient had a normal developmental history since birth, with no past history of chronic medical conditions and no family history of renal problems, malformations, or malignancy.

The patient had been diagnosed with multiple urinary tract infections many times without any radiological investigation. Antibiotics were the main treatment on each occasion. The patient had one child 3 years earlier by normal vaginal delivery at home. During her pregnancy, she complained of two similar episodes and again was diagnosed with urinary tract infection and treated accordingly. She passed her pregnancy period without proper antenatal care and follow-up. She is a housewife of low socioeconomic status and lives in her own house, no history of smoking or alcohol consumption. She had been treated for recurrent urinary tract infections at local clinic with limited resources and without proper investigations.

### Clinical findings

Physical examination at this presentation was normal apart from mild lower abdominal tenderness, but generally the abdomen was soft, and no distention or scar was noted. Clinical observations were unremarkable (Table 1). Her urinalysis showed pus cells of 10–12/high-power field. Renal function blood test results revealed normal urea and serum creatinine levels (Table 2). Urine culture report confirmed positive growth of *Escherichia coli* sensitive only to ciprofloxacin and norfloxacin, and resistant to amoxicillin, erythromycin, tetracycline, and nitrofurantoin.

**Table 1:** Patient’s vital signs at time of admission

Pulse	75 beats per minute
Blood pressure	110/78
Temperature	37 °C
Respiratory rate	19
Oxygen saturation	97%
National Early Warning Score	0

**Table 2:** Laboratory investigation

Test	Value	Normal range
Hemoglobin	129 g/L	120–150 g/L
White cell count	14.5 × 10^9^/L	4–10 × 10^9^/L
Platelets	215 × 10^9^/L	150–410 × 10^9^/L
Neutrophils	11.8 × 10^9^/L	2.0–7.0 × 10^9^/L
Sodium	130 mmol/L	133–146 mmol/L
Potassium	4.5 mmol/L	3.5–5.3 mmol/L
Urea	7.6 mmol/L	2.5–7.1 mmol/L
Creatinine	87 µmol/L	53–115 µmol/L
Estimated glomerular filtration rate	72 mL/min	60–120 mL/min

**Fig. 1:**
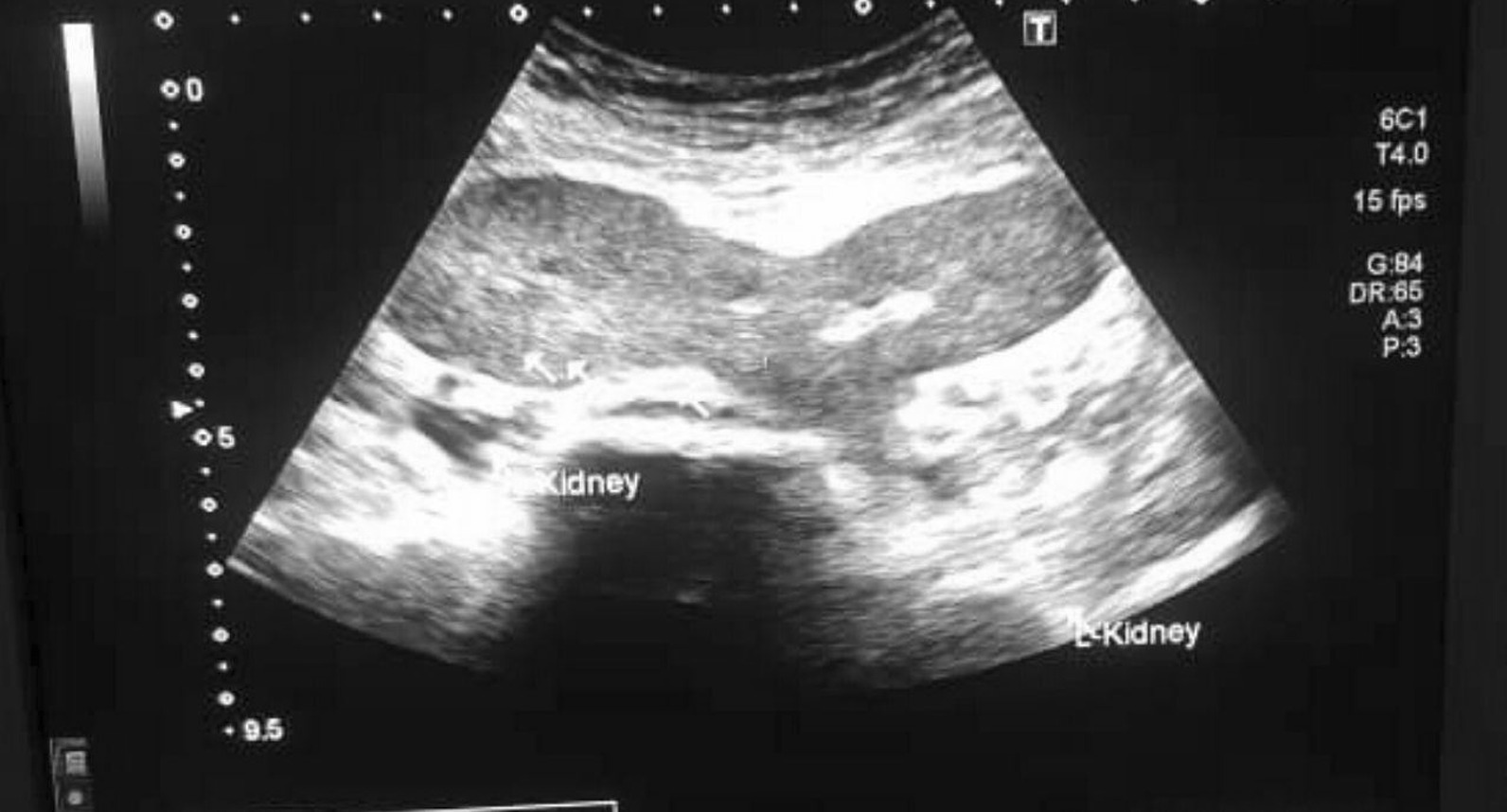
Abdominopelvic ultrasound scan showed ectopic kidneys at the hemi-pelvis, fused in their upper poles, normal size and texture of the kidneys with normal corticomedullary differentiation, no stones or obstructive changes

Ultrasound of the abdomen and pelvis was performed and revealed ectopic kidneys at the hemi-pelvis, fused in their upper poles. Normal size and texture of the kidneys was noted, with normal corticomedullary differentiation. No stones or obstructive changes were found (Fig. 1).

**Fig. 2:**
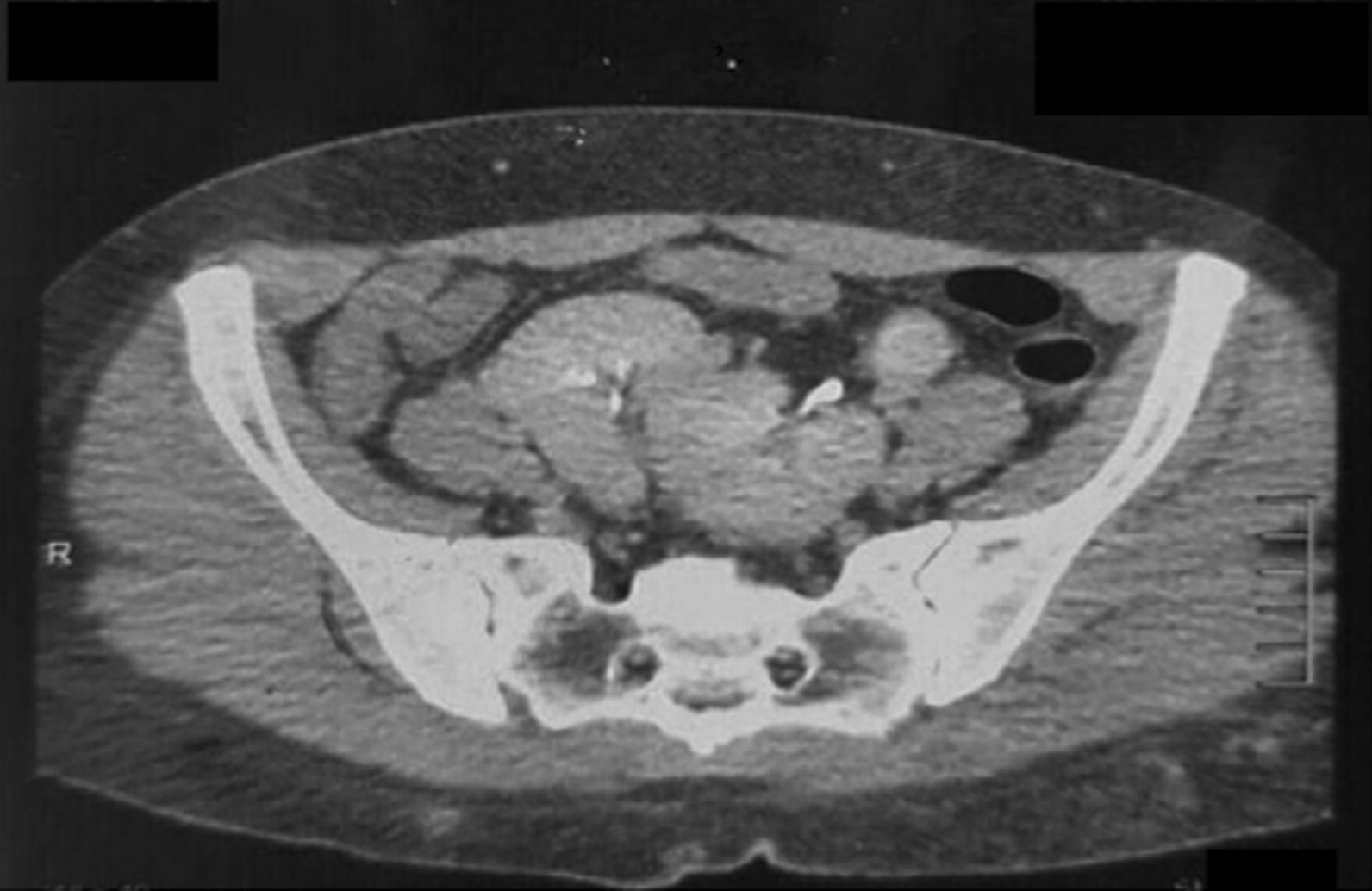
Computed tomography urography. The right kidney is ectopically placed in the pelvis, measures 9.6 cm bipolar length, and is medially and inferiorly faced. The left kidney is also ectopically placed in the pelvis, measures 9.3 cm in bipolar length, is mal-rotated as the pelvis faces upward and laterally, both kidneys are partially fused at their upper poles

**Fig. 3:**
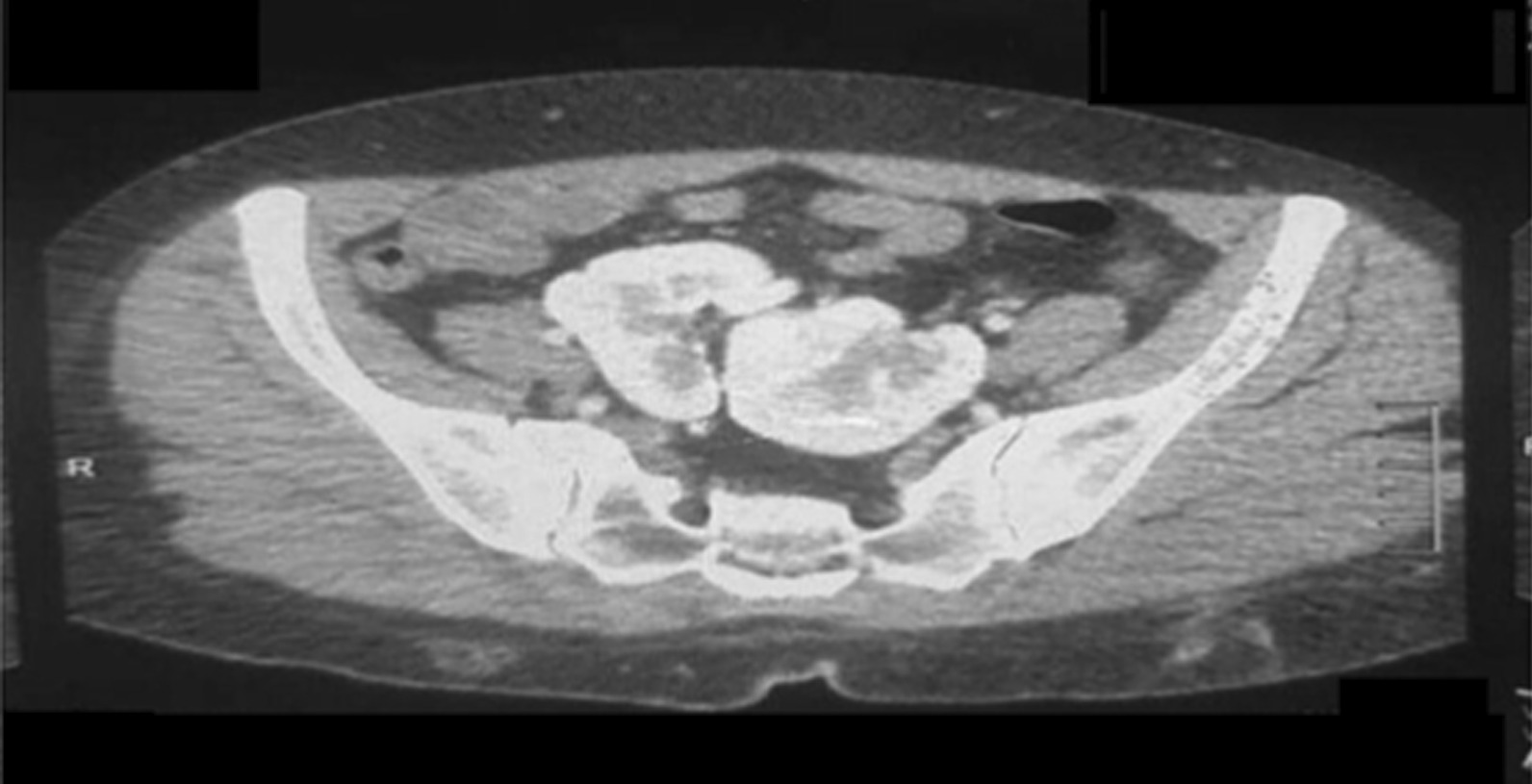
Computed tomography urography. The right kidney is ectopically placed in the pelvis, measures 9.6 cm bipolar length, and is medially and inferiorly faced. The left kidney is also ectopically placed in the pelvis, measures 9.3 cm in bipolar length, and is mal-rotated as the pelvis faces upward and laterally, both kidneys are partially fused at their upper poles

**Fig. 4:**
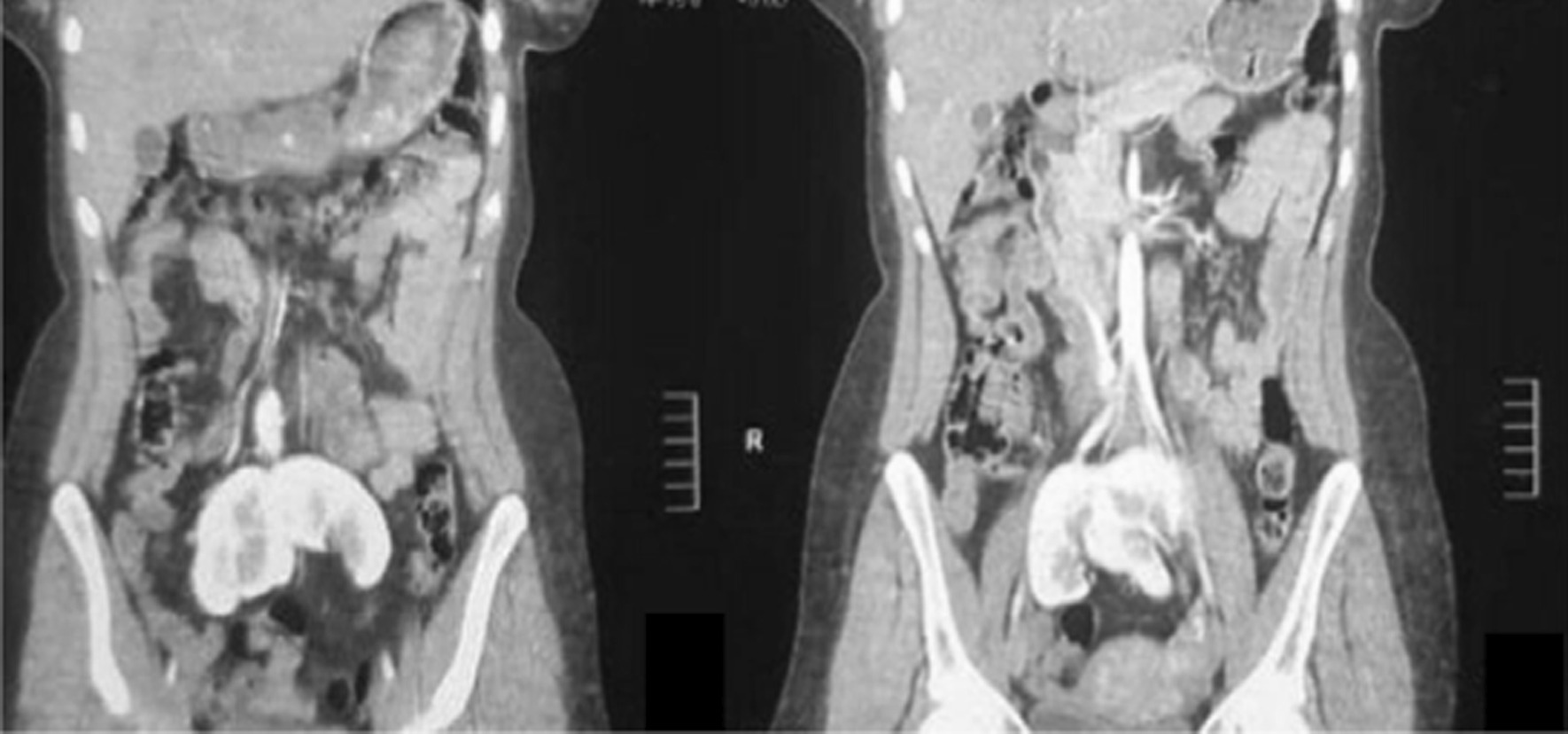
Computed tomography urography. The right kidney is ectopically placed in the pelvis, measures 9.6 cm bipolar length, and is medially and inferiorly faced. The left kidney is also ectopically placed in the pelvis, measures 9.3 cm in bipolar length, and is mal-rotated as the pelvis faces upward and laterally, both kidneys are partially fused at their upper poles

As a part of the radiological workup, computed tomography (CT) urography was performed. This revealed a right kidney ectopically placed in the pelvis, measuring 9.6 cm in bipolar length, medially and inferiorly faced. The left kidney was also ectopically placed in the pelvis, measuring 9.3 cm in bipolar length. It was mal-rotated as the pelvis faced upward and laterally, both kidneys were partially fused at the upper poles (Figs. 2, 3, 4). The report concluded that bilateral ectopic partially fused kidneys in their upper pole were present with the left kidney mal-rotated.

Appropriate antibiotics (ciprofloxacin 400 mg) were given intravenously according to the culture and sensitivity. Outpatient follow-up with repeat ultrasound scan every 3 month was scheduled. At 1-year follow-up, the patient had no further symptoms. Her follow-up course is shown in Table 3.

**Table 3:** summary of patient’s follow-up

Time	Action	Outcome
January 2019	Hospital base assessment (urine culture/USS and CT scan)	Diagnosis of pelvic fused kidneys with chronic UTI. Good response to proper antibiotic treatment
March 2019	Outpatient follow-up, urine general, USS, and home advice	No evidence of infection and no new changes in USS
June 2019	Outpatient follow-up, urine general, USS, and home advice	No evidence of infection per and no new changes in USS
September 2019	Outpatient follow-up, urine general, USS, and home advice	No evidence of infection and no new changes in USS
January 2020	Loss to follow-up and connection difficulties	Patient stopped attending follow-up

*USS* ultrasound scan, *CT* computed tomography, *UTI* urinary tract infection

## Discussion

To the best of our knowledge, this is the first case describing this rare type of renal fusion with reverse malrotation in an adult. Although horseshoe kidney is a not uncommon renal anomaly, the site of fusion is usually at the lower pole of the kidneys, and upper pole fusion is extremely rare. However, the association between renal collecting system abnormality and upper pole fusion is uncommon but cannot be totally excluded. Recurrent history of urinary tract infection represents a classical mode of presentation in such cases. This abnormality usually remains asymptomatic; hence, detection during the early life stage is extremely challenging.

Renal fusion anomaly usually occurs during embryogenesis. Fusion of the kidneys in the lower pole (horseshoe kidney) occurs in 1 per 400 live births [5]. In many cases renal fusion anomaly is associated with renal malrotation either unilaterally or bilaterally [5, 7–9]. Bilateral pelvic kidneys with upper pole fusion and malrotation is considered a very rare type of renal malformation reported in the literature. Early diagnosis and effective therapeutic methods help to prevent the risk of end-stage renal failure from this rare type of renal fusion [10].

Congenital renal anomalies are a rare condition, representing only 10% of all anomalies, with male gender predominance [11]. Ahmad reported fused renal ectopia in a female with complex medical and developmental comorbidities including thrombocytopenia and absent radius syndrome (TAR) [12]. However, we demonstrated abnormal fusion in the upper pole of the kidneys with reverse rotation in a previously fit and well adult female patient with no complex medical comorbidity leading to or linked to this abnormality. Although pelvic fused kidneys can be part of a syndrome such as Müllerian agenesis or unicornuate uterus in females, or can be a feature of multisystem congenital syndromes such as CHARGE syndrome or TAR syndrome, the abnormality can also be an isolated condition without any precipitating factors [13, 14].

Ectopic fused kidneys may mimic symptoms in of acute abdominal or pelvic inflammatory diseases. Consequently, radiological investigations should also be obtained to exclude other pathology [15, 16]. Furthermore, acute and chronic renal infection in preexisting malformed kidneys associated with free fluid or abscess formation can be misinterpreted in radiological scans as renal fistula or even renal cystic tumors, which may lead to unnecessary surgical interventions [17].

Management of recurrent urinary tract infections without ultrasonography or alternative radiological assessment can be challenging and may result in mistaken diagnoses. While ultrasound is limited in its ability to give clear anatomical details about complex renal malformation, it can readily detect renal ectopia early in life. CT scan is the mainstay of renal imaging provided that standardized injection protocols are followed and renal function is assessed. The detection of complex reverse malformation of the kidneys and their anatomical association is possible; however, concerns remain regarding the risk of increased radiation exposure in pediatric patients [11, 18]. As a result, magnetic resonance urography is widely used for complex urinary tract abnormalities and is considered the best imaging technique for different age groups, as it allows for better separation of the renal poles and accurate calculation of renal function [19].

In most cases, renal anomaly is associated with abnormal insertion of the ureter into the bladder, resulting in vesicoureteral reflux. This is usually discovered late, particularly if the renal anomaly is only diagnosed in adulthood [18]. Long-standing vesicoureteral reflux results in serious renal complications such as chronic pyelonephritis, renal abscess, and renal scar formation, and eventually end-stage renal failure [20]. However, in our case, there was no evidence of vesicoureteral reflux despite the presence of reverse malrotation.

The relation between renal ectopia and malignancy is not yet clear; however, some reports have raised concerns about possible increased incidence of tumors in ectopic kidneys [21, 22]. Lifelong follow-up with ultrasound every 6 months or annual CT scans is recommended for such complex urinary tract malformation.

Treatment options vary according to the presence of complications and their severity. Generally, if no vesicoureteral obstruction or deterioration of renal function is observed, then no further treatment is required. Surgical intervention may be indicated when significant renal obstruction or injury is encountered [23].

Health systems in low-income countries differ from those in high-income countries in terms of the availability of resources and access to services. Loss to follow-up among patients with complex problems is a significant challenge facing health care providers in developing countries, with implications for management outcome [24]. Lack of infrastructure and early detection, and financial limitations are the main problems faced in the follow-up of these patients.

## Conclusion

Renal anomalies should be suspected in any adult patient with a recurrent history of urinary tract infections. Ultrasonography should be performed as an initial investigation for all patients with such a history. Early detection of renal malformation is important to minimize the risk of developing stones, end-stage renal failure, and other renal disorders. Magnetic resonance urography is considered the optimal imaging technique in all age groups. This should be undertaken should abnormalities be detected on other imaging modalities, as it allows for better separation of renal poles and accurate calculation of renal function.

## Data Availability

The data used in this report are available to readers.
